# ST14 syndromic epidermal differentiation disorder: A case report of a homozygous recessive variant with photosensitivity

**DOI:** 10.1016/j.jdcr.2026.01.037

**Published:** 2026-02-02

**Authors:** Nermeen Elhofy, Sara Khalid Almukhaimar, Sarah Yousef Alyaseen, Ali Wasel Aldandan, Mohammed Abdulmohsen Al-mulhem

**Affiliations:** a Section of Dermatology, Outpatient Department, Prince Saud Bin Jalawy Hospital, Ministry of Health, Al-Ahsa, Saudi Arabia; b Dermatology, Almoosa Specialist Hospital, Almoosa Health Group, Al-Ahsa, Saudi Arabia; c General Practitioner, King Faisal University, Al-Ahsa, Saudi Arabia; d Pathology, Al-Ahsa Health Cluster, Al-Ahsa, Saudi Arabia

**Keywords:** autosomal recessive ichthyosis, congenital ST14-syndromic epidermal differentiation disorder, hypotrichosis, photophobia, photosensitivity, ST14 gene mutation, ST14-sEDD, syndromic ichthyosis

## Introduction

Hereditary epidermal differentiation disorders (EDDs), formerly known as inherited ichthyosis, are rare conditions characterized by abnormal skin scaling and thickening due to defective cornification.^1^

Syndromic EDDs (sEDDs) may present with additional features, including hair abnormalities, neurological involvement, transient neonatal respiratory distress, and hearing impairment.^2^^,^^3^ ST14-sEDD is an autosomal recessive variant caused by missense mutations in ST14, which encodes matriptase (a type II transmembrane serine protease essential to the matriptase-prostasin proteolytic cascade). Loss of this cascade disrupts filaggrin processing, epidermal lipid extrusion, and stratum corneum desquamation.^4^ Patients typically present with scaling, hyperkeratosis, and hypotrichosis (sparse, brittle, woolly hair), alongside blepharitis, hypohidrosis, and follicular atrophoderma.^3^

While hereditary EDDs are usually diagnosed at birth, complex forms like ST14-sEDD may present subtly, delaying diagnosis. Distinguishing these cases from similar dermatologic conditions requires thorough evaluation. Here, we present a 29-year-old female with ST14-sEDD, highlighting the ongoing diagnostic and therapeutic challenges of inherited ichthyosis.

## Case report

A 29-year-old female patient presented with generalized scaly skin, eye photosensitivity, hypohidrosis, and hypotrichosis. While born with normal skin, her symptoms emerged at 2 months of age. She has no other medical conditions. There is a positive family history of a similar condition on her father’s side. The patient was born to consanguineous parents, who are second cousins, yet both parents are asymptomatic.

**Fig 1:**
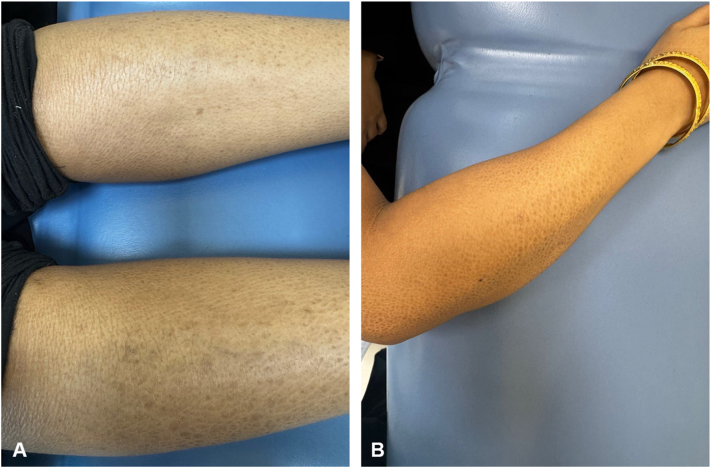
Clinical presentation of diffuse scaling on the arm **(A)** and legs **(B)**. Scales are diffuse, light brown to gray, fine to medium, adherent, and generalized, but more prominent on the extensor surface of the extremities.

**Fig 2:**
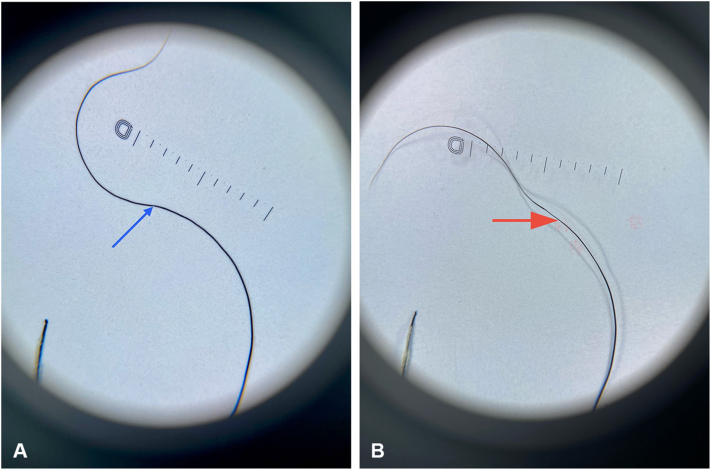
Dermoscopic features of Pili Torti. **A,** The *blue arrow* indicates twisting of the hair shaft on its axis (180 degrees). **B,** The *red arrow* shows flattening of the hair shaft.

**Fig 3:**
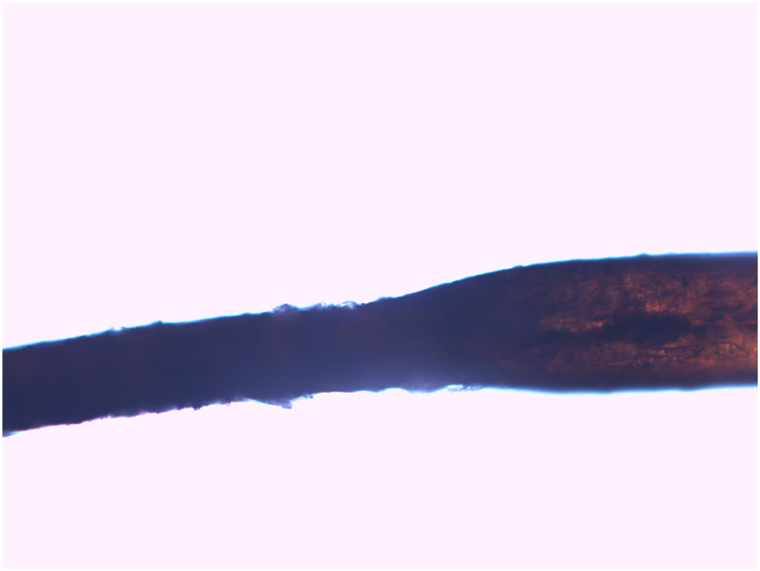
Light microscopy findings of Pili Torti. Light microscopy of the hair shaft reveals both flattening and torsion. The surface of the hair shows focal cuticular disruption, and the medulla appears compressed and irregular at the site of torsion. Original magnification, ×200.

Her physical examination revealed generalized large, fine scales that spared the flexural regions (Fig 1, *A* and *B*). Her scalp hair was sparse, brittle, and woolly, with the Pili Torti sign evident under both dermoscopic examination (Fig 2, *A* and *B*) and light microscopy (Fig 3). Additionally, she had sparse eyebrows and absent eyelashes, accompanied by generalized hypotrichosis affecting axillary and pubic hair; however, other secondary sexual characteristics were normal. She also experienced photophobia and had a refractive error. Her teeth and nails appeared normal.

Laboratory tests revealed a vitamin B12 deficiency, with a level of 147 pg/mL compared to the normal range of 200-900 pg/mL. Additionally, mild anemia was detected, evidenced by hemoglobin at 12 g/dL, where the normal range is 12-16 g/dL, and a low ferritin level of 24 ng/mL, with a normal range of 30-200 ng/mL. Genetic testing, conducted through whole exome sequencing, identified a positive ichthyosis panel. A homozygous variant was found in the ST14 gene, consistent with autosomal recessive ST14-associated ichthyosis, also known as ST14-sEDD.

The patient underwent a comprehensive supportive regimen, including regular use of emollients and keratolytics – specifically, white paraffin, 10% urea, and salicylic acid preparations – for skin care. Hair involvement was treated with 2% topical minoxidil and weekly use of ketoconazole shampoo. Ocular dryness was managed with artificial tears. Furthermore, the patient received oral cholecalciferol (vitamin D3) 10,000 IU once weekly, ferrous sulfate 190 mg every other day, and vitamin B12 (Mecobalamin) injections 500 mcg once weekly to address associated deficiencies.

## Discussion

Although ichthyosis typically presents at birth, our patient's symptoms emerged at 2 months of age – later than most reported ST14-sEDD cases, which often show significant flexural involvement from birth.^3^, ^4^, ^5^, ^6^ In contrast, her manifestations persisted through adolescence with flexural sparing, prompting further investigation that eventually confirmed the diagnosis.

The differential diagnosis for an adult presenting with lifelong ichthyosis, hypotrichosis, and photophobia includes various forms of autosomal recessive congenital ichthyosis, such as those involving TGM1, NIPAL4, ALOX12B, ALOXE3, and CYP4F22. While these forms infrequently present with the combination of hypotrichosis, pili torti, hypohidrosis, and photophobia observed in the patient, they remain relevant considerations. Trichothiodystrophy is another possibility, as it can manifest with ichthyosis, photosensitivity, brittle and sparse hair, and neurological features; however, the characteristic low-sulfur “tiger-tail” banding was absent in this case. Additionally, ichthyosis-hypotrichosis-sclerosing cholangitis should also be considered. Ichthyosis-hypotrichosis-sclerosing cholangitis shares symptoms such as ichthyosis and hypotrichosis but is generally associated with additional conditions like sclerosing cholangitis, dental anomalies, and scarring alopecia. These were not present in this case. Similarly, Netherton syndrome was considered unlikely due to the absence of trichorrhexis invaginata and atopy.^2^^,^^7^ Thus, considering these features collectively, the most consistent diagnosis is autosomal recessive ichthyosis with hypotrichosis related to ST14.

Genetic analysis identified a mutation in exon 11 of ST14, consistent with previously documented cases.^4^^,^^5^ However, the phenotypic variability observed here underscores the clinical heterogeneity of this condition.

Photosensitivity is an underrecognized feature of the ST14-sEDD spectrum. Our patient experienced ocular photophobia, corroborating recent literature of ocular involvement in ST14-sEDD.^3^ A previously described newborn with a confirmed ST14 pathogenic variant exhibited severe photophobia with tearing, preventing him from keeping his eyes open outdoors.^6^ Our patient's milder presentation further illustrates phenotypic variability and suggests photophobia may be an underreported rather than rare finding, warranting further investigation into ocular manifestations of ST14 mutations.

Nutritional deficiencies – including selenium, iron, vitamin D, and zinc – have been linked to ichthyosis severity.^8^ Our patient had low vitamin B12 and ferritin levels, which may have contributed to her cutaneous manifestations. Targeted supplementation could offer clinical benefit.

## Conclusion

Significant advancements in understanding the genetics underlying ichthyosis syndromes have been made. However, the variability in clinical presentation, as observed in this patient, underscores the need for further research. It is essential to delineate the phenotype and comprehend the full spectrum of pathology associated with ST14-sEDD. Additionally, there is a need for more targeted therapies to provide long-term relief.

## Conflicts of interest

None disclosed.
